# Les vertiges et la perte auditive chez le personnel de santé atteint de COVID-19

**DOI:** 10.11604/pamj.2024.48.65.31375

**Published:** 2024-06-25

**Authors:** Bahri Ghada, Ben Said Hanene, Siwar Chemingui, Hiba Ziedi, Najla Mechergui, Nizar Ladhari

**Affiliations:** 1 Service de Pathologie Professionnelle et de Médecine du Travail, Hôpital Charles Nicolle, Tunis, Tunisie

**Keywords:** COVID-19, vertige, sensation vertigineuse, perte d’audition, symptômes neurologiques, COVID-19, vertigo, dizziness, hearing loss, neurologic symptom

## Abstract

La maladie à coronavirus 2019 (COVID-19) affecte le système respiratoire. L´étude des caractéristiques cliniques de cette infection a mis en évidence son tropisme au système nerveux qui serait responsable des atteintes neurologiques et sensorielles, en particulier les vertiges et la perte auditive. Déterminer la fréquence et les caractéristiques de l´atteinte neurologique représentée par les vertiges et la baisse de l´acuité auditive chez les professionnels de santé (PS) atteint de la COVID-19. Etude descriptive transversale menée auprès des PS de l´Hôpital Charles Nicolle (HCN) de Tunis atteints de COVID-19 durant la période allant de septembre 2020 à décembre 2020. La collecte de données a été réalisée par un suivi téléphonique régulier de l´état de santé de ces PS pendant la période d´isolement sanitaire. Un total de 482 PS atteints de COVID-19 ont été colligés. L´âge moyen de la population était de 41 ± 10 ans, dont 111 hommes (23%) et 371 femmes (77%). Les principales manifestations neurologiques étaient: les céphalées (71,2%), l´anosmie (60%), les vertiges (21,8%) et l´hypoacousie (1,5%). Les patients présentant des vertiges étaient significativement plus âgés (P=0,035), de sexe féminin (P=0,003), obèses (P=0,014), souffrant de comorbidités (P=0,004) et ayant une ancienneté professionnelle plus importante (P=0,009). Les vertiges étaient significativement associés à la fièvre (P=0,001), aux douleurs abdominales (P=0,001) et à la désaturation (P=0,039). Les symptômes neurologiques dont les vertiges et la perte auditive pourraient être le seul signe avec lequel on pourrait reconnaitre un cas de COVID-19. La sensibilisation à une telle présentation des patients COVID-19 est cruciale pendant cette période de pandémie pour prévenir la propagation infectieuse.

## Abstract (en)

Coronavirus disease 2019 (COVID-19) affects the respiratory system. Studying the clinical characteristics of this infection has revealed its tropism to the nervous system, which is responsible for neurological and sensory damage, in particular, dizziness and hearing loss. To determine the frequency and characteristics of the neurological impairment represented by dizziness and hearing loss in healthcare professionals (HCP) with COVID-19. Cross-sectional descriptive study conducted among HCP at Charles Nicolle Hospital (CNH) in Tunis affected by COVID-19 during the period from September 2020 to December 2020. Data collection was carried out by regular telephone follow-up of COVID-19 symptoms in these HCPs during the period of sanitary isolation. A total of 482 HCPs with COVID-19 were collected. The average age of the population was 41 ± 10 years, of which 111 were men (23%) and 371 were women (77%). The main neurological manifestations were: headache (71.2%), anosmia (60%), dizziness (21.8%), and hearing loss (1.5%). Patients with vertigo were significantly older (P=0.035), female (P=0.003), obese (P=0.014), suffering from more comorbidities (P=0.004), and having greater professional seniority (P=0.009). Dizziness was significantly associated with fever (P=0.001), abdominal pain (P=0.001), and desaturation (P=0.039). Neurological symptoms including dizziness and hearing loss may be the only sign with which a case of COVID-19 could be recognized. Raising awareness of such a presentation of COVID-19 patients is crucial during this pandemic period to prevent infectious spread, especially in hospitals.

## Introduction

En décembre 2019, un nouveau coronavirus, hautement pathogène nommé SARS-CoV-2, est apparu à Wuhan en Chine et s´est rapidement propagé dans le monde entier. Le 26 juillet 2021, l´Organisation mondiale de la Santé (OMS) a signalé 193798265 cas confirmés de COVID-19, dont 4158041 décès [1]. Ce virus est à l´origine d´un syndrome respiratoire aigu sévère. Les symptômes de la maladie à coronavirus 2019 (COVID-19) peuvent apparaître entre 2 et 14 jours après l´exposition. Néanmoins, des cas d´infection asymptomatique ont été observés [2]. Les symptômes typiques de la maladie sont la fièvre, une toux sèche, une dyspnée, des céphalées et une pneumonie [3]. La COVID-19 affecte les systèmes respiratoire et cardiovasculaire, mais également le système nerveux central (SNC) et périphérique (SNP) [4]. Certains patients peuvent ne pas présenter de symptômes typiques de la COVID-19 et signalent uniquement des manifestations neurologiques dont la plupart surviennent au début de la maladie [5]. Au service de médecine du travail de l´Hôpital Charles Nicolle (HCN) de Tunis, une «unité COVID-19» dédiée à la prise en charge des professionnels de la santé (PS) atteints de COVID-19 a été créée. Cette unité permettait la prise en charge du PS du diagnostic positif de l´infection à la reprise du travail après la maladie. Afin de suivre de près l´évolution du tableau clinique de la COVID-19 et garantir un retour précoce au travail, un suivi téléphonique régulier a été envisagé. La symptomatologie rapportée était polymorphe, nous avons constaté des manifestations oto-rhino-laryngées de type vertiges et une baisse de l´acuité auditive. Ces symptômes ont été également rapportés dans la littérature et considérés comme des manifestations non spécifiques du SNC. Toutefois, peu d´études aussi bien internationales que nationales ont porté sur les présentations neurologiques atypiques et possibles de cette nouvelle infection virale. Aussi, une information et une sensibilisation de la population, en particulier des professionnels de la santé, à la possibilité d´une atteinte neurologique précoce de la COVID-19, est nécessaire afin de réduire précocement, le risque de transmission virale dans les établissements de soins, milieu de travail particulièrement exposant.

Dans ce cadre, nous avons mené une étude à l´HCN de Tunis auprès des PS COVID-19 positifs, qui avait pour objectif de déterminer la fréquence et les caractéristiques de l´atteinte neurologique représentée par les vertiges et la baisse de l´acuité auditive chez les PS atteints de COVID-19.

## Méthodes

Il s´agissait d´une étude descriptive, transversale menée à l´HCN de Tunis, ayant intéressé les PS atteints de la maladie COVID-19 pendant la période allant de septembre à décembre 2020 et répondant aux critères d´éligibilité cités ci-après.

Ont été inclus dans l´étude tous les PS de l´HCN atteints de la COVID-19 durant la période d´étude, dont l´infection était confirmée par un prélèvement virologique, ayant été prise en charge à «l´unité COVID-19» et bénéficiant d´un suivi téléphonique régulier pendant la période du confinement. Les externes en médecine, les stagiaires en soins infirmiers et les PS ne travaillant pas à l´HCN n´étaient pas inclus dans l´étude. Les PS n´ayant pas répondu au suivi téléphonique réalisé durant la période de confinement ont été exclus.

Les données ont été recueillies à partir des dossiers médicaux des PS, à partir d´une fiche de suivi préétablie employée lors du suivi téléphonique et d´un questionnaire préétabli administré à la visite médicale de reprise. Ce questionnaire comportait des données sociodémographiques, professionnelles et médicales. Le suivi téléphonique a été réalisé par les médecins travaillant dans le service de Médecine du Travail de l´hôpital pendant la période du confinement sanitaire, à partir de la date de confirmation de la maladie jusqu´à la reprise de travail (à raison d´un appel téléphonique tous les deux jours). Ce suivi a permis d´identifier la nature des symptômes rapportés notamment neurologiques et sensorielles (les vertiges et la perte auditive en particulier) avec la surveillance de leur évolution dans le temps durant la période d´isolement.

Les données recueillies ont été saisies et analysées par le logiciel SPSS 25. Les fréquences et les pourcentages ont été calculés pour les variables qualitatives et les moyennes et les écarts-types pour les variables quantitatives. Nous avons utilisé le test de l´analyse de la variance (ANOVA) pour la comparaison des moyennes en analyse univariée et le test de chi-deux de Pearson ou Fisher exact pour la comparaison des pourcentages. Le seuil de significativité a été fixé à 5%.

## Résultats

**Figure 1:**
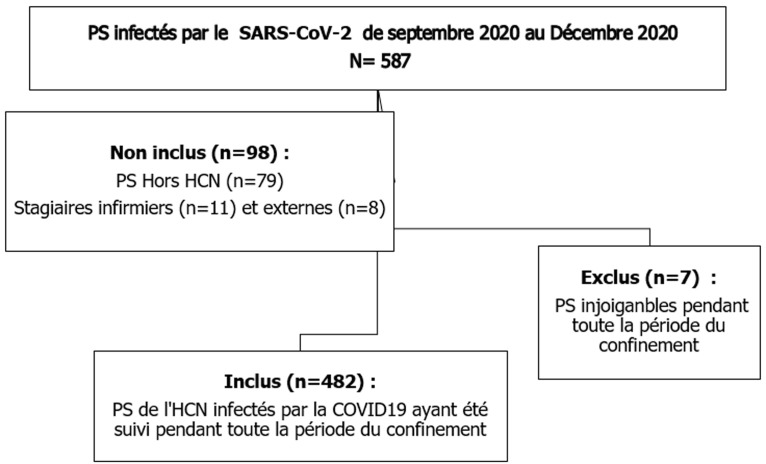
diagramme de flux de la constitution de la population d´étude

Durant la période d´étude 587 cas de COVID-19 parmi les PS ont été confirmés par un prélèvement pour RT-PCR. Un total de 482 PS (82,1%) a été inclus dans notre étude (Figure 1). L´âge moyen de la population était de 41 ± 10 ans. Elle était répartie en 111 hommes (23%) et 371 femmes (77%). La catégorie professionnelle la plus représentée était celle des infirmiers (32,1%) et la plupart des PS travaillaient dans des services à activité chirurgicale (36,5%) (Tableau 1). Le motif de consultation du service de Médecine du Travail pour la majorité des PS (94%) était l´apparition des symptômes. Les symptômes les plus fréquemment rapportés étaient la fièvre (188 cas (39%)), la toux (335 cas (69,5%)) et l´asthénie (393 cas (81,5%)). La durée moyenne de l´évolution des symptômes était de 12,3 ± 5,5 jours.

**Tableau 1:** relation entre la présence de vertiges et d´hypoacousie et les caractéristiques démographiques et professionnelles de la population d´étude

	Absence de vertiges et/ou d’une hypoacousie (n=371)	Présence de vertiges et/ou d’une hypoacousie (n=111)	Total (n=482)	
**Sexe**				**NS**
Homme	95 (25.6%)	16 (14.4%)	111 (23 %)	
Femme	276 (74.4%)	95 (85.6%)	371 (77%)	
Indice de masse corporelle (Kg/m^2^)	28	27.1 ± 5	26.8 ± 4.5	NS
Ancienneté professionnelle (ans)	13	15.6	13,7	0.047
Age de diagnostic (année)	40 ± 10 ans	42.5 ± 10.5 ans	41 ± 10 ans	NS
**Catégorie professionnelle**				
Médecin stagiaire	64 (17.3%)	4 (3.6%)	68 (14.1%)	
Médecin titulaire	21 (5.7%)	1 (0.9%)	22 (4.5%)	
Infirmier	118 (31.8%)	37 (33.3%)	155 (32.1%)	
Technicien	88 (23.7%)	31 (28%)	119 (24.7%)	
Ouvrier	46 (12.4%)	27 (24.3%)	73 (15.1%)	
Femme de ménage	6 (1.6%)	2 (1.8%)	8 (1.6%)	
Agent administratif	25 (6.7%)	9 (8.1%)	34 (7%)	
Aide-soignant	3(0.8%)	0	3 (0.6%)	
**Catégorie du service**				
Service de médecine aigue (réanimation/urgences)	45 (12.1%)	9 (8.1%)	54 (11.2%)	
Services médicaux	130 (35%)	34 (30.6%)	164 (34%)	
Services chirurgicaux	128 (34.5%)	48 (43.2%)	176 (36.5)	
Services généraux	29 (7.8%)	8 (7.2%)	37 (7.7%)	
Laboratoire	22 (6%)	10 (9%)	32 (6.6%)	
Administration	17 (4.6%)	2 (1.8%)	19 (4%)	

Les principales autres manifestations d´origine neurologique rapportées par notre population étaient représentées par: les céphalées (71,2%), l´anosmie (60%), les vertiges (21,8%) et l´hypoacousie (1,5%). Les troubles auditifs tels que l´otalgie et les bourdonnements d´oreille étaient rapportés respectivement par 4 et 5 patients. La durée médiane d´évolution des céphalées était de 4 jours. L´hypoacousie apparait en moyenne vers le dixième jour après le début des symptômes. La durée moyenne d´apparition des vertiges était de 5,85 ± 4,3 jours avec une apparition moyenne vers le neuvième jour après le début des symptômes. Les vertiges ou l´hypoacousie ont été les premiers signes évoqués par les PS atteints de signes neurologiques dans 14.4% (N=16) des cas.

L´âge moyen des PS présentant des vertiges ou une baisse de l´acuité auditive était de 43 ± 9 ans avec un sex ratio de 0,16. Les PS présentant des vertiges étaient significativement plus âgés (P=0,035), de sexe féminin (P=0,003), obèses (P=0,014) souffrant plus de comorbidités (P=0,004) et ayant une ancienneté professionnelle plus importante (P=0,009) que les patients ne rapportant pas de vertiges. La durée moyenne d´évolution des symptômes chez les PS souffrant de vertiges était de 14.3 ± 6.7 jours mais aucune association statistique significative n´a été démontrée entre cette durée et la présence des vertiges. Les vertiges étaient significativement associés à la fièvre (P=0,001), aux douleurs abdominales (P=0,001) et à la désaturation (P=0,039). Un traitement symptomatique a été administré pour les vertiges dans 26,7% des cas. Une hypoacousie associée à des vertiges a été notée chez un PS et associée à d´autres troubles auditifs tels que l´otalgie et les bourdonnements d´oreille chez cinq PS (1%). Le Tableau 2, résume les caractéristiques fondamentales des patients atteints de COVID-19 présentant ou non des vertiges.

**Tableau 2:** les caractéristiques cliniques des patients atteints de COVID-19 avec ou sans vertiges

Variables	Nombre de patients			P
	Absence des vertiges	Présence des vertiges	Total	
Antécédents pathologiques	189 (50.4%)	72 (69 %)	261 (54.1%)	0.004
Diabète	31 (8.2%)	9 (8.6%)	40 (8.3%)	NS
Hypertension	33 (87.5%)	17 (16.2%)	50 (10.4%)	NS
Asthme	38 (10%)	10 (9.5 %)	48 (10%)	NS
BPCO	4 (1%)	4 (3.8 %)	8 (1.7%)	NS
Néoplasie	6 (1.6%)	1 (1%)	7 (1.5%)	NS
Cardiopathie	15 (4%)	7 (6.7%)	22 (4.6%)	NS
Néphropathie	8 (2%)	2 (2%)	10 (2%)	NS
Maladies psychiatriques	29 (7.7%)	11 (10.5%)	40 (8.3%)	NS
Troubles ostéoarticulaires	27 (7.2%)	22 (21%)	49 (10.2%)	<10^-3^
Maladies de système	5 (1.3%)	3 (2.9%)	12 (2.5%)	NS
Tabagisme	41 (10.9%)	6 (5.7%)	47 (9.8%)	NS
Activité sportive	134 (35.5%)	26 (24.8%)	160 (33.2%)	NS
**Symptômes**				
Fièvre	132 (35%)	56 (53.3%)	188 (39%)	0.001
Toux	253 (67.1%)	82 (78.1%)	335 (69.5%)	NS
Asthénie	298 (79%)	95 (90.5%)	393 (81.5%)	NS
Anosmie	220 (58.4%)	68 (64.8%)	288 (59.7%)	NS
Dyspnée	89 (23.6%)	65 (62%)	154 (32%)	NS
Douleurs thoraciques	61 (16.2%)	59 (56.2%)	120 (24.9%)	NS
Céphalées	252 (66.8%)	91 (86.7%)	343 (71.1%)	NS
Diarrhée	178 (47.2%)	74 (70.5 %)	252 (52.3%)	NS
Nausées et vomissements	41 (10.9%)	14 (13.3 %)	55 (11.4%)	NS
Douleurs abdominales	21 (5.6%)	25 (23.8 %)	64 (13.3%)	0.001
Durée totale des symptômes	12 ± 5.2 jours	14.3 ± 6.7jours	12.3 ± 5.5 jours	NS
Hospitalisation	8 (2%)	6 (5,7%)	14 (3%)	NS
Désaturation	20 (5.3%)	7 (6.7%)	27 (5,6%)	0.039
Oxygénothérapie à domicile	11 (3%)	0 (0%)	11 (2,3%)	NS
Total	377	105	482	

BPCO: Bronchopneumopathie chronique obstructive

## Discussion

Nous avons été intéressés par l´étude des caractéristiques cliniques de l´atteinte neurologique représentée par les vertiges et la baisse de l´acuité auditive chez 482 PS atteints de COVID-19. La population d´étude était jeune (âge moyen de la population était de 41 ± 10 ans) avec une prédominance féminine de 77%. Les principales manifestations neurologiques étaient: les céphalées (71,2%), l´anosmie (60%), les vertiges (21,8%) et l´hypoacousie (1,5%). Les patients présentant des vertiges étaient significativement plus âgés (P=0,035), de sexe féminin (P=0,003), obèses (P=0,014), souffrant de comorbidités (P=0,004) et ayant une ancienneté professionnelle plus importante (P=0,009). Les vertiges étaient significativement associés à la fièvre (P=0,001), aux douleurs abdominales (P=0,001) et à la désaturation (P=0,039).

Cette étude peut être considérée parmi les premières études tunisiennes évaluant l´atteinte neurologique chez les patients COVID-19 positifs. L´effectif de la population serait également important comparativement aux autres études menées sur le plan national ou international [6,7]. L´étude avait pour objectif de déterminer la fréquence et les caractéristiques de l´atteinte neurologique en rapport avec la COVID-19, représentée en particulier par les vertiges et la baisse de l´acuité auditive chez les professionnels de la santé (PS) de l´Hôpital Charles Nicolle (HCN) de Tunis. Toutefois, le recueil de données basé sur le contact téléphonique conférait une part de subjectivité dans la description des manifestations neurologiques, en particulier les vertiges et les pertes auditives rapportées par les PS infectés.

Dans notre population, 21,8% des cas et 1,5% avaient présenté respectivement des vertiges et une baisse de l´acuité auditive. En effet, l´infection par le virus du SARS-CoV-2 peut revêtir plusieurs aspects cliniques dont l´aspect le plus classique est une pneumonie. Cependant, avec l'évolution de la pandémie et l'accumulation des cas, il était démontré que des manifestations neurologiques peuvent être des manifestations cliniques de l'infection par le SARS-CoV-2. Les nouveaux cas de troubles de l´odorat et du goût incitaient à la possibilité d´un effet neuropathique du SARS-CoV-2 [8]. Semblables aux effets des virus sur les voies neuronales du sens olfactif, les vertiges post-viraux ou la perte auditive sont des séquelles connues des virus pouvant provoquer une névrite vestibulaire ou une labyrinthite [9]. Ces manifestations peuvent parfois précéder les caractéristiques typiques du COVID-19 comme la fièvre et la toux qui se développeront plus tard [10]. Les vertiges sont aussi les manifestations non spécifiques du SNC les plus fréquemment rapportées par les malades atteints de la COVID-19 [5]. Par ailleurs, la COVID-19 est considérée comme la conséquence d´une mutation du virus du syndrome respiratoire aigu sévère et du virus du syndrome respiratoire du Moyen-Orient. Ces virus ne sont pas principalement des virus neurotropes et leur principale cible est l'épithélium respiratoire [10].

L'entrée du SARS-CoV-2 dans les cellules hôtes humaines est principalement médiée par le récepteur cellulaire de l'enzyme de conversion de l'angiotensine 2 (ACE 2), qui est exprimé dans l'épithélium des voies aériennes, le parenchyme pulmonaire, l'endothélium vasculaire, les cellules rénales et les cellules intestinales [11]. Les récepteurs ACE 2 sont également présents dans le tissu neuronal [12]. En plus des récepteurs ACE 2, l'invasion directe, l'hypoxie, l'hypercoagulopathie, ainsi que la réaction immunologique faisaient partie des mécanismes de l´atteinte neurologique provoquant des vertiges [13]. Les céphalées et les vertiges sont également considérés comme des symptômes non spécifiques et mineurs de nombreuses maladies. Ils ont été signalés comme des symptômes associés à la présentation de la COVID-19 dans différentes études. Leur incidence variait entre 3 et 12,1% [10]. Dans une série de cas publiée dans le *Journal of American Medical Association (JAMA)* de patients atteints de COVID-19 à Wuhan, en Chine, les symptômes neurologiques étaient fréquents. Cette étude était menée dans 3 centres de soins spéciaux désignés pour la COVID-19, auprès de 214 patients hospitalisés dont 88 (41,1%) avaient une infection grave et 126 (58,9%) avaient une infection non grave [5]. Environ 36,4% des patients présentaient des manifestations du système nerveux impliquant le SNC (24,8%), le SNP (8,9%) et les muscles squelettiques (10,7%). Chez les patients présentant des manifestations du SNC, les symptômes les plus fréquemment rapportés étaient des vertiges (16,8%) et des céphalées (13,1%). De plus, les manifestations du système nerveux étaient significativement plus fréquentes dans les infections graves par rapport aux infections non graves (45,5% vs 30,2%, P = 0,02) [5].

Ce constat pourrait nous inciter à être plus vigilant dans le suivi des patients révélés malades qui se présentent avec des symptômes d´origine neurologique afin de prévenir l´évolution vers les formes graves. Selon une revue de la littérature incluant 14 articles, un total de 141 patients étaient regroupés. Tous ces patients présentaient des vertiges comme symptôme de présentation de COVID-19. Les vertiges étaient la présentation initiale de COVID-19 chez 3/141 patients, soit 2.13%, dont deux développaient plus tard une symptomatologie respiratoire [6]. Malayala *et al*. rapportaient le cas d´une jeune femme présentant des vertiges sévères associés à des nausées et à des vomissements sans autres signes typiques évocateurs de COVID-19. L´infection au COVID-19 était suspectée et confirmée par un prélèvement pour RT-PCR devant la contamination massive par le SARS-CoV-2 dans son environnement professionnel. Le diagnostic de névrite vestibulaire induite par la COVID-19 était retenu et une rééducation vestibulaire était prescrite avec une nette amélioration clinique [14]. Ce cas suggère l'importance de penser à la COVID-19 chez les patients présentant des signes neurologiques notamment en absence de signes respiratoires ou en cas de signes respiratoires retardés. Kong *et al*. rapportaient un premier cas de pneumonie induite par le SARS-CoV-2 qui commençait par une atteinte isolée du système nerveux [15].

Concernant la baisse de l´acuité auditive, certaines observations suggéraient l´envahissement des voies neuronales impliquées dans l´audition par le SARS-CoV-2. En effet, les virus provoquent le plus souvent une perte auditive neurosensorielle et la récupération de l´audition peut parfois se produire spontanément [16]. Notre travail notait sept cas de PS rapportant la notion de perte auditive. Toutefois, cette hypoacousie n´était pas objectivée par une exploration spécifique auditive constituant ainsi une limite de l´étude. Les PS étaient, par ailleurs, adressés à la consultation d´Oto-Rhino Laryngologie (ORL) après la fin de leur isolement. Une étude récente chez des patients asymptomatiques atteints de COVID-19 révélait une augmentation des seuils de son pur à haute fréquence tandis que les émissions oto-acoustiques évoquées transitoires étaient considérablement réduites, le tout en l'absence de symptômes otologiques [16]. Une revue de la littérature, publiée en avril 2021, incluait un total de 7 articles dont 3 études transversales et 4 rapports de cas. Au total, 35 patients âgés de 20 à 60 ans étaient inclus. Tous les patients inclus avaient une perte auditive comme symptôme principal et les symptômes qui l'accompagnaient étaient des acouphènes, des vertiges et des otalgies. Les tests auditifs les plus couramment utilisés étaient l'audiométrie tonale, la tympanométrie et l'émission otoacoustique [7].

Cette revue signalait que la COVID-19 peut se présenter par une perte auditive isolée. L´étude de Kilic *et al*. notait un cas de perte auditive neurosensorielle soudaine chez un jeune de 29 ans sans antécédents pathologiques particuliers. La surdité de perception unilatérale était le seul symptôme présent chez ce patient [17]. Par ailleurs, la présentation initiale d'une infection par le SARS-CoV-2 avec otalgie et acouphènes était aussi rapportée [18]. Fidan *et al*. rapportaient le cas d´une surdité de transmission isolée secondaire à une otite moyenne aiguë [18]. Cependant, il est à noter que dans l´étude de Degen *et al*. l'implant cochléaire était réalisé sous anesthésie locale chez un patient qui a développé un surdité neurosensorielle soudaine [19]. Au vu de ces études, nous soulignons que la perte auditive peut être l´une des manifestations cliniques de l´infection par le SARS-CoV-2, notamment la perte auditive neurosensorielle.

Afin d´étudier les caractéristiques des vertiges et de la baisse de l´acuité auditive, une corrélation était étudiée entre ces symptômes et les caractéristiques sociodémographiques, professionnelles et cliniques. Une corrélation positive était notée entre les vertiges et l´âge avancé, le sexe féminin, l´obésité, les antécédents de comorbidités comme les troubles ostéoarticulaires ainsi que la désaturation. Nos résultats concordaient avec ceux d´autres études qui ont mis en évidence une association entre les vertiges et les différents paramètres relevés, pouvant expliquer l´apparition des vertiges chez ce personnel féminin âgé et présentant certaines comorbidités, bien qu´étudiés comme manifestation neurologique du COVID-19. En effet, dans l´étude de Mao *et al*. les patients atteints d'une infection sévère, plus susceptibles de présenter des manifestations neurologiques, étaient significativement plus âgés et présentant plus de comorbidités, en particulier l'hypertension [5]. Aussi, les symptômes de déséquilibre ou de vertige augmentent en fréquence et en sévérité avec l´avancée en âge et sont plus fréquents chez les femmes [20]. Le vertige positionnel paroxystique bénin, pouvant être de cause virale, était également associé à l'âge avancé, au sexe féminin, au diabète, et à l´ostéoporose et à l´intubation [21,22].

Au terme de notre travail, il s´avère que les vertiges et la baisse de l´audition peuvent se présenter parmi les symptômes d´origine neurologique, évocateurs de la COVID-19. Ils doivent être ainsi recherchés systématiquement, aussi bien au début de l´investigation de la maladie que pendant le suivi des cas qui se sont avérés positifs.

## Conclusion

Notre travail a conclu à une possibilité d´une présentation de la COVID-19 par des manifestations d´origine neurologique comme les vertiges et la baisse de l´audition bien que leur fréquence soit peu élevée. Ces symptômes non spécifiques pourraient être le seul signe avec lequel on pourrait reconnaitre un cas de COVID-19. Ainsi, la sensibilisation et l´information des professionnels de soins d´une telle présentation non spécifique de la COVID-19 sont cruciales pendant cette période de pandémie afin de prévenir la propagation infectieuse et afin d´initier précocement un traitement ciblé COVID-19 permettant de limiter l´évolution vers des formes graves.

### Etat des connaissances sur le sujet

- *A l´origine, la COVID-19 était présentée comme une maladie du système respiratoire affectant le parenchyme pulmonaire dont les symptômes prédominants étaient représentés par la fièvre, la toux et l´essoufflement;*
- *Aujourd´hui, le tableau clinique incluait des symptômes neurologiques divers notamment les vertiges et la perte auditive;*
- *Les vertiges et l´hypoacousie ont été décrits dans la plupart du temps comme des séquelles de la COVID-19 et non pas comme des signes cliniques de la phase aiguë de l´infection*.

### Contribution de notre étude à la connaissance

- *Notre travail a mis en évidence une prévalence considérable des symptômes neurologiques notamment les vertiges et la perte auditive pendant la phase aiguë de l´infection;*
- *Ces symptômes peuvent être annonciateurs de la COVID-19, leur détection est un moyen de dépistage chez les professionnels de santé;*
- *Ceci suggère l'importance de penser à la COVID-19 chez les professionnels de santé présentant des vertiges ou une hypoacousie même en absence de signes respiratoires*.
